# Years of Life Lost Due to Complete Suicide in Iran: A National Registry-Based Study

**DOI:** 10.34172/jrhs.2024.140

**Published:** 2024-03-18

**Authors:** Mehran Rostami, Abdollah Jalilian, Mohammad Jalilian, Seyed Amirhosein Mahdavi

**Affiliations:** ^1^Deputy of Health, Kermanshah University of Medical Sciences, Kermanshah, Iran; ^2^Department of Statistics, Razi University, Kermanshah, Iran; ^3^Department of Clinical Sciences, Faculty of Veterinary Medicine, Garmsar Branch of Islamic Azad University, Semnan, Iran; ^4^Legal Medicine Research Center, Legal Medicine Organization, Tehran, Iran

**Keywords:** Burden of disease, Cause of death, Life expectancy, Mortality, Self-harm

## Abstract

**Background:** Suicide was the fourth leading cause of death among individuals aged 15 to 29 years worldwide in 2019, highlighting its significant impact on young people. Iran’s suicide-related mortality rate was 5.1 per 100000 population in the same year, which is lower than the global average. This study aimed to estimate the years of life lost (YLLs) due to complete suicide in Iran.

**Study Design:** A registry-based cross-sectional study.

**Methods:** The data on complete suicide cases used in this study were obtained from the national suicide registry of the Iranian Forensic Medicine Organization (FMO) that was registered between March 21, 2016 and March 20, 2020.

**Results:** The total number of YLL due to premature death by suicide over the four-year period was 611068 years (15.97 per 1000 persons) in males, 286847 years (7.65 per 1000 persons) in females, and 897915 years (11.86 per 1000 persons) for both genders. Moreover, the age group of 15–29 years experienced the highest YLL attributed to suicide. Furthermore, the study revealed an increasing trend of YLL due to suicide among individuals aged 30–44.

**Conclusion:** These findings highlight the significant impact of suicide on the loss of potential years of life in Iran. The study indicates that the young and productive age groups of 15-29 and 30-44 years are particularly affected, with the highest YLL due to complete suicide. The study provides valuable insights for designing targeted and evidence-based suicide prevention programs that can reduce the burden of suicide in Iran, particularly among young and middle-aged adults.

## Background

 Suicide (intentional self-harm) is a serious public health problem and remains one of the leading causes of death globally.^1^ It imposes substantial economic and social costs, causing loss of productivity and adverse effects on the mental health of the affected individuals, their social networks, and family members.^2^ Suicide disproportionately affects young people, with suicide being the fourth leading cause of death among individuals aged 15-29 years worldwide in 2019.^1^ In the same year, around 760 000 deaths by suicide were reported worldwide, with an age-standardized mortality rate of 9.0 per 100 000 population in both genders globally.^2^ In Iran, the suicide-related mortality rate was 5.1 per 100 000 population in 2019, which is lower than the global average.^2^ One way to measure the impact of suicide is by estimating the years of life lost (YLLs) due to premature death caused by suicide.^3^ The YLL due to suicide is a crucial metric for comprehending the burden of suicide and the potential impact of suicide prevention efforts.^3^ Thus, the YLL due to suicide among young people represents a substantial loss of productive years and potential societal contributions.^1,4^ Understanding the burden of suicide in Iran is essential in developing effective suicide prevention strategies and improving mental health interventions.^4^

 Policies driven only by mortality rates must be revised to achieve allocative efficiency with limited resources as they fail to account for morbidity, disease category, cost-effectiveness, health perception, and decision-making. Therefore, relying solely on the number of deaths may not provide sufficient information about the YLL associated with a health event. Mortality rates provide incomplete information as they do not reveal the age distribution of deaths or how risk levels vary with age.^5^ The YLL due to a disease can be influenced by various factors, including mortality rates and the age distribution of deaths.^5^ A higher YLL may be attributed to higher mortality or deaths occurring at younger ages or both.^5^ The YLL is easier to comprehend and translates into actionable policy interventions, making it a valuable contribution to public health research and policy.

 In Iran, limited research has been conducted on the YLL due to suicide.^3,6,7^ This study thus aimed to estimate the YLL due to complete suicide in Iran. The study’s findings can inform targeted suicide prevention efforts, leading to more effective measures for preventing suicide and reducing the burden of complete suicide in Iran.

## Methods

###  Study population

 Iran is a country located in the Middle East. It is bordered by Iraq to the west, Turkey to the northwest, Azerbaijan, Armenia, Turkmenistan, and the Caspian Sea to the north, Afghanistan to the east, Pakistan to the southeast, and the Persian Gulf to the south. Iran had over 86.5 million people in 2021, making it the world’s 17^th^ most populous country, and it covers an area of around 1.64 million km^2^.

###  Source of suicide data

 According to Iranian regulations, all sudden or unexpected deaths, including those resulting from intentional self-harm, must be reported to the Forensic Medicine Organization (FMO) for forensic investigation.^8^ Forensic physicians and pathologists examine these cases and issue a death certificate specifying the exact cause of death. All cases referred to the FMO undergo forensic autopsy as part of the process.^9^ If the forensic examination confirms that the cause of death was intentional self-harm, the case is included in the national suicide registry maintained by the FMO.^9^ Consequently, the suicide registration data from the FMO’s database are considered the most comprehensive source for complete suicide data in Iran.^8^ This is due to Iranian legal directives assigning that causes of death be exclusively written in Persian on death certificates issued by forensic physicians, with ICD codes or English terms prohibited.^9^ For this cross-sectional study, data on age, gender, method, and year of death of people who committed suicide were extracted from the FMO’s suicide registry database. The study analyzed suicide data and covered a specific period from March 21, 2016 to March 20, 2020. This timeframe was selected based on the availability of suicide mortality data provided to the authors by Iran’s FMO.

###  Source of life expectancy data

 The standard life expectancy values used for Iran in calculating YLLs were extracted from the Global Burden of Disease (GBD) studies conducted by the Institute for Health Metrics and Evaluation (IHME). These values are a reference for estimating the number of years lost due to premature death in different age groups for both genders in each study year.^10^ According to the World Health Organization (WHO) estimates in Iran, life expectancy at birth improved to 77.3 years in 2019.^11^ To estimate the number of YLL and to enable international comparisons, age groups should be categorized in a way that the age group closest to the highest life expectancy is considered the last age group. Monaco with the highest life expectancy at 87 years followed by Japan at 86 years was considered the golden standard. Therefore, in this study, the last age group was 85 and above, so individuals older than life expectancy have not lost any years due to premature mortality.

###  Statistical analysis

 In the GBD, the calculation of YLLs in the reference case is based on a simplified methodology that excludes age-weighting and discounting.^12^ This simplified approach was based on the WHO guidelines for calculating the number of YLL. It relied on the sum of each death multiplied by the standard life expectancy associated with each age group in which death occurs. This equation allowed for estimating the number of years individuals lose due to premature death. The specific equation used for this calculation was as follows^13^:


$$YLL=\sum N_{x}\times L_{x}$$

Where *N*_x_ represents the number of deaths at each age group *x*, *L*_x_ indicates the standard life expectancy at that age group *x*, and the summation (∑) in the formula runs over the range of x, which represents each 5-year age group. By utilizing this approach, the reference case aimed to provide a more transparent and straightforward assessment of the burden of disease, without assigning differential weights to younger age groups or discounting future health outcomes.

 In the initial calculation of YLLs, age groups were defined as follows: 5–9, 10-14, 15–19, 20–24, 25–29, 30–34, 35–39, 40–44, 45–49, 50–54, 55–59, 60–64, 65–69, 70–74, 75–79, 80–84, and ≥ 85. YLLs were computed for each age group using the earlier formula. However, to align with previous studies,^3,6^ the calculated YLLs were integrated and reconstructed into 10-to-15-year age intervals. This step was taken to ensure consistency and comparability with earlier research efforts. The reconstructed age groups used for further analysis were as follows: 5–14, 15–29, 30–44, 45–59, 60–74, 75–84, and ≥ 85. The age group closest to the higher life expectancy is referred to as the oldest age group. When making temporal or spatial comparisons, it is important to control for the population size. This can be achieved by dividing the YLL by the population at a specific time or in a specific region. This division helps to standardize the measure, taking into account differences in population size (per 1000 people).^14^ Population estimates by age groups and gender were extracted from the statistical yearbook of Iran,^15^ and all calculations were performed in Microsoft Excel 2021 software.

 We also computed the average annual percentage change (AAPC) to determine the summary measure of trend over the entire time interval.^16^ AAPC is calculated based on the Joinpoint model for the whole observed timeline,^16^ and the analysis for the trend was performed using Joinpoint Regression Program version 4.6.0.0.

## Results

 This cross-sectional study recorded 19 268 complete suicide cases from March 21, 2016 to March 20, 2020 (based on Persian calendar time). Of these cases, 70.9% and 29.1% were men and women, respectively, and the median age of these individuals was 32 years. The distribution of suicides across the study period was 22.8% in the first year, 24% in the second year, 26.5% in the third year, and 26.7% in the final year. Overall, at the national level, the most common suicide method was hanging (51.6%) followed by self-poisoning (26.8%), with an observable difference from other methods. Tables 1 and 2 present more characteristics of people who committed suicide by age- and gender-specific, as well as suicide methods.

**Table 1:** Characteristics of people committing suicide between March 2016 and March 2020 based on gender and suicide methods

**Time**	**Male**	**Female**	**Total**	**Hanging**	**Self-** **poisoning**	**Self-** **immolation**	**Firearm**	**Jumping** **from Height**	**Others**^a^
March 21, 2016- March 20, 2017	3163	1238	4401	2322	1123	286	286	185	199
March 21, 2017- March 20, 2018	3265	1358	4623	2362	1206	327	292	245	191
March 21, 2018- March 20, 2019	3609	1492	5101	2673	1383	263	276	272	234
March 21, 2019- March 20, 2020	3626	1517	5143	2587	1449	305	286	268	248

^a^ Wrist cutting, cold weapon, drowning, suffocation, other specified means, and unusual suicide methods.

**Table 2:** Age-specific and gender-specificcharacteristics of people committing suicide based on methods between March 2016 and March 2020

**Age-groups (y)/ Gender**	**Suicide method**					
	**Hanging**	**Self-poisoning**	**Self-** **immolation**	**Firearms**	**Jumping** **from Height**	**Others** ^a^
5-14						
Male	167	15	2	9	5	2
Female	125	44	7	4	20	3
Both	292	59	9	13	25	5
15-29						
Male	2825	1336	143	570	179	210
Female	1103	927	291	99	230	98
Both	3928	2263	434	669	409	308
30-44						
Male	2759	1061	228	249	204	252
Female	549	674	236	16	110	70
Both	3308	1735	464	265	314	322
45-59						
Male	1368	517	86	108	77	126
Female	241	244	98	4	52	26
Both	1609	761	184	112	129	152
60-74						
Male	482	179	25	56	45	53
Female	110	81	42	2	22	12
Both	592	260	67	58	67	65
75-84						
Male	122	47	12	14	18	13
Female	24	16	7	0	2	3
Both	146	63	19	14	20	16
≥ 85						
Male	58	19	4	9	5	4
Female	11	1	0	0	1	0
Both	69	20	4	9	6	4
Total						
Male	7781	3174	500	1015	533	660
Female	2163	1987	681	125	437	212
Both	9944	5161	1181	1140	970	872

^a^ Wrist cutting, cold weapon, drowning, suffocation, other specified means, and unusual suicide methods.

 As a reminder, we employed WHO guidelines in calculating the burden of complete suicide. Table 3 provides information on the age-specific and gender-specific numbers of deaths and YLL due to suicide reported from the Iranian FMO based on the study period. The distribution of the total number of YLL across the study period was 205,737 in the first year, 215,934 in the second year, 236 346 in the third year, and 239 898 in the final year. Furthermore, the study revealed an alarming trend of increasing YLL due to suicide among individuals aged 30–44. This observation underscores the need for attention and intervention in addressing suicide prevention in this age group. These findings emphasize the substantial burden of premature mortality caused by suicide, particularly in the age groups of 15–29 and 30–44 years (more details are presented in Table 3). Table 4 presents information on age-specific and gender-specific YLL and YLL rates (per 1000 persons) by suicide methods. In all age groups, the highest number of YLL due to suicide methods was attributed to hanging with a total of 458 772 years. In addition, the highest YLL rate due to suicide methods was attributed to hanging (6.06 per 1000 persons) for both genders. Moreover, the total number of YLL due to premature death by suicide over the four years was 611 068 years (15.97 per 1000 persons) in males, 286 847 years (7.65 per 1000 persons) in females, and 897 915 years (11.86 per 1000 persons) for both genders combined. Furthermore, among all age groups, individuals aged 15–29 years experienced the highest number and proportion of YLL due to suicide. Generally, the highest number of YLL and YLL rates due to suicide methods were attributed to hanging, self-poisoning, firearms, self-immolation, and respectively (more details are presented in Table 4). Based on Joinpoint analysis, the summary measure of trend over the whole observation period was significantly different from zero at the alpha = 0.05 level (AAPC = 4.96, *P* < 0.05).

**Table 3:** Number of deaths and YLL due to complete suicide reported from the Iranian Forensic Medicine Organization between March 2016 and March 2020 based on gender and age groups

**Age** **groups**	**March 21 2016- March 20 2017**						**March 21 2017- March 20 2018**						**March 21 2018- March 20 2019**						**March 21 2019- March 20 2020**					
	**No. of Deaths**			**No. of YLL**			**No. of Deaths**			**No. of YLL**			**No. of Deaths**			**No. of YLL**			**No. of Deaths**			**No. of YLL**		
	**Male**	**Female**	**Both**	**Male**	**Female**	**Both**	**Male**	**Female**	**Both**	**Male**	**Female**	**Both**	**Male**	**Female**	**Both**	**Male**	**Female**	**Both**	**Male**	**Female**	**Both**	**Male**	**Female**	**Both**
5-14	59	49	108	3940	3439.1	7379.1	41	51	92	2742.6	3582.2	6324.8	61	43	104	4099	3027	7126	39	60	99	2622.6	4233.5	6856.1
15-29	1243	610	1853	70195.2	36976.5	107171.7	1270	694	1964	72240	42009	114249	1354	741	2095	77180	45110	122290	1396	703	2099	79795	42918.2	122713.2
30-44	1106	365	1471	48837.3	17225	66062.3	1128	388	1516	50143.3	18157	68300.3	1210	431	1641	53635	20392	74027	1309	471	1780	57813	22208.2	80021.2
45-59	522	137	659	15679	4464.1	20143.1	541	154	695	16419.5	4974.1	21393.6	651	181	832	19877.3	5981.7	25859	568	193	761	17371.3	6286	23657.3
60-74	164	60	224	3083.2	1178	4261.2	199	52	251	3739	1003.2	4742.2	249	80	329	4631	1566.5	6197.5	228	77	305	4275.7	1518.3	5794
75-84	45	13	58	421.3	129.8	551.1	66	16	82	623	162.3	785.3	59	11	70	560	103.7	663.7	56	12	68	540.7	126.5	667.2
≥ 85	24	4	28	145.35	23	168.35	20	3	23	121.3	17.1	138.4	25	5	30	153.2	29.1	182.3	30	1	31	183.5	5.8	189.3
Total	3163	1238	4401	142301.4	63435.5	205736.9	3265	1358	4623	146028.7	69904.9	215933.6	3609	1492	5101	160135.9	76210.2	236346.2	3626	1517	5143	162601.8	77296.5	239898.3

*Note.* No: Number; YLL: Years of life lost.

**Table 4:** Age-specific and gender-specificYLL and YLL rate due to suicide methods reported from the Iranian Forensic Medicine Organization between March 2016 and March 2020

**Age Groups (yr)/ Gender**	**No. of YLL (year)**						**YLL Rate (per 1000 persons)**					
	**Hanging**	**Self-** **poisoning**	**Self-** **immolation**	**Firearms**	**Jumping** **from Height**	**Others**	**Hanging**	**Self-** **poisoning**	**Self-** **immolation**	**Firearms**	**Jumping** ** from Height**	**Others**
5-14												
Male	11,189.3	1,004.5	133.9	602.8	340	133.8	1.67	0.15	0.02	0.09	0.05	0.02
Female	8790	3096	492	281.3	1411.4	211.2	1.38	0.49	0.08	0.04	0.22	0.03
Both	19,979.3	4100.5	625.9	884.1	1751.4	345	1.53	0.31	0.05	0.07	0.13	0.03
15-29												
Male	160,856.7	75,469.5	8130.1	32,904.6	10,220.7	11,979.3	17.33	8.13	0.88	3.54	1.1	1.29
Female	67,463.8	55,856.4	17,427.4	6145.8	14,202.6	5927.5	7.51	6.22	1.94	0.68	1.58	0.66
Both	228,320.5	131,325.9	25,557.5	39,050.4	24,423.3	17,906.8	12.5	7.19	1.4	2.14	1.34	0.98
30-44												
Male	122,113.9	47,128.5	10,066.2	10,994.2	9,046.5	11,078.6	10.49	4.05	0.86	0.94	0.78	0.95
Female	25,954.3	31,660.5	11,118.5	751.1	5146.7	3350.4	2.28	2.79	0.98	0.07	0.45	0.29
Both	148,068.2	78,789	21,184.7	11,745.3	14,193.2	14,429	6.44	3.43	0.92	0.51	0.62	0.63
45-59												
Male	41,516.4	15,797.4	2,621.1	3317.8	2,077.3	3,753	6.25	2.38	0.39	0.5	0.31	0.56
Female	7843.5	8057.3	3186.2	136.2	1644	838.7	1.20	1.24	0.49	0.02	0.25	0.13
Both	49,359.9	23,854.7	5807.3	3,454	3,721.3	4591.7	3.75	1.81	0.44	0.26	0.28	0.35
60-74												
Male	9079.5	3336.3	478	1031	939.1	977.4	2.98	1.09	0.16	0.34	0.31	0.32
Female	2142.7	1563	855.7	45.4	419.6	238.3	0.65	0.48	0.26	0.01	0.13	0.07
Both	11,222.2	4899.3	1333.7	1076.4	1358.7	1215.7	1.77	0.77	0.21	0.17	0.21	0.19
75-84												
Male	1165.5	440.9	111.3	133	176.3	119.6	1.73	0.66	0.17	0.2	0.26	0.18
Female	239.6	158.3	70.5	0	18.9	27.1	0.35	0.23	0.10	0	0.03	0.04
Both	1405.1	599.2	181.8	133	195.2	146.7	1.04	0.44	0.13	0.1	0.14	0.11
≥ 85												
Male	353.7	115.7	24.4	55	30.5	24.3	1.31	0.43	0.1	0.2	0.11	0.09
Female	63.5	5.8	0.0	0.0	5.7	0.0	0.23	0.02	0.0	0.0	0.02	0.0
Both	417.2	121.5	24.4	55	36.2	24.3	0.77	0.22	0.04	0.1	0.07	0.04
Total												
Male	346,275	143,292.9	21,565	49,038.4	22,830.4	28,066	9.05	3.75	0.56	1.28	0.6	0.73
Female	112,497.4	100,397.3	33,150.3	7359.8	22,848.9	10,593.2	3.00	2.68	0.88	0.2	0.61	0.28
Both	458,772.4	243,690.2	54,715.3	56,398.2	45,679.3	38,659.2	6.06	3.22	0.72	0.74	0.6	0.5

*Note.* YLL: Years of life lost; No: Number.

## Discussion

 Population health summary measures such as disability-adjusted life years (DALY) provide a more comprehensive assessment of the burden of disease, accounting for mortality and morbidity. Due to premature mortality, a significant component of DALY, YLLs are an efficient tool for resource allocation, investigating epidemiological associations, and informing public health priorities.^5^ Several methods are developed for estimating YLL. When choosing among various methods for estimating YLL, researchers should consider the research objectives, the available data, and the model’s flexibility.^5^ In Iran, for example, this epidemiological method has been used to evaluate the burden of occupational injury,^17^ homicide mortality,^18^ drug-related deaths,^19,20^ cancer mortality,^21,22^ burn-related accidental deaths,^23^ and the coronavirus disease 2019 (COVID-19).^24^

 A total of 19 268 complete suicide cases were registered in the Iranian FMO database from March 2016 to March 2020. Our findings indicated that men account for approximately 71% of the registered cases of complete suicide. Hanging was the most common method of complete suicide, accounting for over 51% of cases. During the study period, the total number of YLLs due to premature death by suicide was 897 915 for both genders (11.86 per 1000 persons). Among all age groups, individuals aged 15-29 years had the highest number and proportion of YLL due to suicide. Furthermore, the study found an increasing trend in the YLL due to suicide among individuals aged 30-44 years. These findings underscore the importance of targeted prevention and intervention efforts aimed at decreasing the burden of suicide, particularly among individuals aged 15-29 and 30-44 years who have the highest YLL attributed to suicide in Iran. In other words, these findings underscore the substantial burden of premature mortality caused by suicide in Iran. This study highlights the age groups of 15–29 years and 30–44 years as particularly affected, with the highest YLL attributed to suicide. The study’s results emphasize the urgent need for effective suicide prevention and intervention efforts, which can help reduce the human and economic costs of suicide in Iran. By identifying the characteristics of people who committed suicide based on gender and suicide methods associated with YLL, the study provides valuable insights for designing targeted and evidence-based suicide prevention programs that can reduce the burden of suicide in Iran, particularly among young and middle-aged adults.

 As already noted, limited research has been conducted in Iran to investigate the YLL related to complete suicide. At the national level, between 2006-2015, there were 35 297 recorded deaths due to suicide in Iran. Data on suicide-related deaths were obtained from the Iranian FMO. The total YLL during these ten years was 34.5 per 1000 persons for males, 13.6 per 1000 persons for females, and 23.4 per 1000 persons for both genders. Hanging was the leading cause of suicide-related YLL.^3^ Although Iran has a lower suicide mortality rate compared to European countries, the YLLs rate is comparable, that is, death occurs at younger ages in Iran at a similar rate to European countries.^3^ At the sub-national level, the Fars suicide surveillance system provided data that included suicide-related mortality from 2011 to 2018. The study identified 2384 mortalities, with a mean age of 32.7 years. The total YLL for the Fars study were calculated to be 58 669 years, equivalent to 14.7 per 1000 persons.^6^ The study found that regardless of year or gender, suicide contributed to the largest YLL for individuals aged 15-29 years.^6^ At another sub-national level, in Ilam province from 2014 to 2018, suicide-related deaths were included in the study, using data obtained from the Iranian FMO. The total YLL for the Ilam study during this period was estimated to be 15 685, and the YLL rate for suicide was found to be 34.4 for both genders combined.^7^ The study’s findings revealed that men and individuals aged 15-29 contributed more significantly to the YLL.^7^ As it can be observed, the results of our study are consistent with prior studies.

 According to the 2019 WHO estimates, complete suicide represents about 1.3% of all deaths globally.^2^ Despite the decreasing trends recorded in both genders in most countries, including Iran in the Middle East and North Africa during the 2000-2019 period, the age-standardized suicide mortality rate showed an increasing trend in some populations such as Saudi Arabia and Syria.^2^ Interpreting trends requires caution to ensure accuracy and reliability. For example, the WHO estimated 4334 suicide deaths in both genders in 2019.^1^ The *WHO’s* estimates are based on data officially reported by the Ministry of Health (MoHME). As we know, national death registration coverage (percentage) of Iran’s MoHME was close to 90% in 2019.^25^ Therefore, the national death registration system in Iran has an underreporting. Based on data from the Iranian FMO, the present study reports a higher number of cases, exceeding 5100. The discrepancy in recorded suicide deaths across different sources may have significant implications for estimating suicide trends, potentially resulting in lower data quality. The discrepancy between the WHO estimate and the number of cases reported in the present study emphasizes the significance of using consistent data sources and exercising caution in interpreting suicide trend analyses. Further research is thus necessary to clarify the true extent of the problem and improve data quality to ensure accurate estimates of suicide trends. Previous findings would suggest that to decrease suicide and enhance societal mental health effectively, policy-makers should implement practical strategies, including promoting social equity, advocating for modest living, expanding family counseling centers, empowering women, and addressing conflicts arising from modern-traditional value clashes.^26-28^ These measures can significantly reduce suicide incidence in Iran.

 Considering the conservative context of Iranian society, suicide data encounters diverse limitations. The social stigma associated with suicide leads to the concealment of such deaths within families. For example, in self-immolation deaths, the victim’s intent is sometimes attributed to accidents rather than deliberate acts.^28^ Consequently, accurate categorization becomes challenging, potentially leading to underestimating self-immolation deaths and overestimating burn-related accidental deaths. In addition, where the cause of death is categorized as suicide within the under-five age group, it indicates a misclassification commonly referred to as a garbage code within the death registry.^29^ It is essential to exclude the total population from being categorized as at risk for suicide. Handling the total population as an at-risk category might lead to underestimating suicide rates.^29^ Regarding the data collection form used by the Iranian FMO for suicide cases, there is no item specifically addressing the pregnancy status of women at the time of suicide, as well as the time interval between delivery and suicide. This indicates that the correlation between postpartum depression and complete suicide has not been adequately assessed in Iran.^30^

**Highlights**

- The total number of years of life lost (YLLs) due to premature death by suicide was 897 915 years for both genders combined during the period from March 2016 to March 2020.
- The age group of 15–29 years experienced the highest number and proportion of YLL attributed to suicide.
- The study emphasizes the specific impact on young and productive age groups, with the highest YLL due to complete suicide.
- Based on Joinpoint analysis, the summary measure of trend over the whole observation period is significantly different from zero at the alpha = 0.05 level (AAPC = 4.96).

## Conclusion

 These findings highlight the significant impact of suicide on the loss of potential years of life in Iran. The study reports that the young and productive age groups of 15-29 and 30-44 years are particularly affected, with the highest YLL due to complete suicide. The study also provides valuable insights for designing targeted and evidence-based suicide prevention programs that can reduce the burden of suicide in Iran, particularly among young and middle-aged adults.

## Acknowledgments

 The authors wish to thank Mr. Rasoul Najafi (Ph. D student in Biostatistics, Yazd University of Medical Sciences, Yazd, Iran) for his useful comments and technical support in the preparation of the paper.

## Authors’ Contribution

**Conceptualization:** Mehran Rostami.

**Data curation:** Seyed Amirhosein Mahdavi.

**Formal analysis:** Mehran Rostami, Abdollah Jalilian.

**Investigation:** Seyed Amirhosein Mahdavi.

**Methodology:** Abdollah Jalilian.

**Project administration:** Mehran Rostami.

**Supervision:** Seyed Amirhosein Mahdavi.

**Validation:** Mehran Rostami.

**Visualization:** Mehran Rostami.

**Writing–original draft:** Mehran Rostami, Abdollah Jalilian, Mohammad Jalilian, and Seyed Amirhosein Mahdavi.

**Writing–review & editing:** Mehran Rostami, Mohammad Jalilian, and Seyed Amirhosein Mahdavi.

## Competing Interests

 In this study, the corresponding author (Dr. Mahdavi) is one of the vice-chancellors of the Iranian Legal Medicine Organization. Other authors have no conflict of interests.

## Ethical Approval

 The study protocol was reviewed and approved by the Ethics Committee of the Legal Medicine Organization (reference number: IR.LMO.REC.1401.017). No informed consent was required for this study due to the anonymized and de-identified nature of the suicide-related mortality data.

## Funding

 The authors received no financial support for this research, authorship, and/ or publication.
